# Intervention to Improve Preschool Children’s Fundamental Motor Skills: Protocol for a Parent-Focused, Mobile App–Based Comparative Effectiveness Trial

**DOI:** 10.2196/19943

**Published:** 2020-10-20

**Authors:** E Kipling Webster, Chelsea L Kracht, Robert L Newton Jr, Robbie A Beyl, Amanda E Staiano

**Affiliations:** 1 Institute of Public and Preventive Health Augusta University Augusta, GA United States; 2 Pennington Biomedical Research Center Baton Rouge, LA United States

**Keywords:** children, technology, family, motor skills, physical activity

## Abstract

**Background:**

Preschool age is an important time to master fundamental motor skills (FMS) through structured physical activity, yet many young children lag behind in motor skill development.

**Objective:**

The Promoting Lifelong Activity in Youth (PLAY) study is a pilot comparative effectiveness trial to test the acceptability, feasibility, and preliminary effectiveness of a mobile app delivered to parents to promote FMS development in their preschool children (aged 3-5 years).

**Methods:**

We conducted a 2-arm, parallel-design, randomized comparative effectiveness trial in 72 parent-child dyads from the southeastern United States. Experts in motor development and developmental psychology developed an app designed to deliver a 12-week program to parents of preschoolers using 1 of 2 curricula: an FMS program (intervention) that involved peer modeling, parent engagement, and structured skills-based activities and an unstructured physical activity (comparator) curriculum that provided suggestions for child-led physical activity (ie, free play). Primary outcomes are feasibility and acceptability of the app and child’s FMS measured at end of intervention (week 12). Exploratory outcomes are child’s objective physical activity, perceived movement competence, and parent report of self-regulation at the end of treatment (week 12) and sustained outcomes at follow-up (week 24).

**Results:**

This project was funded in September 2018, with institutional review board approval in August 2018. Data collection took place from May 2019 through February 2020. To date, the project team has completed data collection on 69 preschool-age children, and results are expected to be published by 2021.

**Conclusions:**

The PLAY study examines the feasibility and preliminary effectiveness of a mobile app, parent-led curricula to promote FMS proficiency for preschool children. If found to be effective, the app has the potential for wide-scale dissemination to parents of preschoolers and to provide a model for the utilization of mobile apps to promote young children’s motor skill development.

**Trial Registration:**

ClinicalTrials.gov NCT03901300; https://clinicaltrials.gov/ct2/show/NCT03901300

**International Registered Report Identifier (IRRID):**

DERR1-10.2196/19943

## Introduction

### Background

Physical activity (PA) is vital to early childhood physical and mental development [1]. Yet a third of children aged 3-5 years do not engage in the recommended 3 hours of daily PA [2]. Structured PA promotes the development of fundamental motor skills (FMS) like running, jumping, or throwing a ball. These skills are basic, goal-directed movement patterns that provide a foundation for children to be physically active and competent movers [3] and enable a child to build the confidence and ability to be physically active [3,4]. These skills are not innately acquired and instead must be modelled and practiced for mastery. Evidence has shown that children must establish a minimal level of FMS proficiency to continue being physically active as they age [4-8].

FMS and PA behaviors have a dynamic and reciprocal relationship [4,7]. Children with higher levels of FMS are more physically active both during childhood [9-11] and into adolescence [12-15] compared to those with lower levels of FMS competency. Research has indicated that the preschool years are an opportune time for children to learn and reinforce these skills [16]. With inadequate FMS competency, a child is less likely to engage in PA based on a lack of prerequisite skills and abilities that are foundational to FMS [4-8], whereas a child with adequate FMS competency tends to be more physically active [17]. Moreover, FMS promote self-regulatory abilities including managing emotions, focusing attention, and inhibiting behavior [18]. Behavioral self-regulation is important for academic readiness [19] and regulating health behaviors that may also contribute to obesity [20]. Therefore, interventions for preschool children should focus on developing FMS competence.

As children and their parents spend a significant amount of time viewing screens [21], it may be opportune to leverage screen-time as a tool to increase preschool children’s PA. Specifically, PA interventions delivered over digital devices, such as mobile apps, can provide encouragement in real-world settings for children to be physically active. The use of mobile-based interventions (eg, on a smartphone or tablet) has been recognized as a promising avenue to substantially affect levels of PA, especially in high-income countries such as the United States [22]. However, few mobile-based interventions have been developed that specifically focus on increasing PA in children [23,24], while several weight management apps include PA as a secondary component [25,26].

Comparable mobile health (mHealth) programs that focus on increasing PA in preschoolers are promising: A 6-month mobile app intervention delivered to parents of preschoolers improved children’s PA, particularly among those with higher fat mass [27]. A 7-week intervention that involved cognitive behavioral skills training over text messaging to parents of preschoolers observed improved PA in the target child, but this intervention also included face-to-face visits with a counselor [28]. Only one of these studies delivered the intervention exclusively to parents, and the PA results were not reported [29]. In a slightly older age group (6-10 years of age), a mobile-based PA promotion program (P-Mobile) was delivered to parents and resulted in increased objectively measured steps/day [30]. The present intervention, the Promoting Lifelong Activity in Youth (PLAY) study, is focused on modeling FMS as a way to increase preschool-aged children’s FMS, PA levels, perceived motor competence, and academic readiness.

### Study Aims

The goal of PLAY is to test a developmentally appropriate intervention delivered on a mobile app to parents, with the goal of teaching FMS proficiency to their preschool-aged children (aged 3-5 years). The specific aims are described in the following sections.

#### Aim 1

The first aim is to examine the feasibility and acceptability of a 12-week FMS intervention delivered through a mobile app to parents and children.

#### Aim 2

The second aim is to test the hypothesis that a 12-week FMS intervention delivered through a mobile app will improve children’s FMS, compared to the unstructured PA (UPA) app comparator group.

#### Exploratory Aim 1

The first exploratory aim is to test the hypothesis that a 12-week FMS intervention delivered through a mobile app will improve children’s PA levels, perceived movement competence, and academic readiness (ie, self-regulation skills), compared to the UPA app comparator group.

#### Exploratory Aim 2

The second exploratory aim is to test the potential mediating or moderating effect of FMS on changes in exploratory outcomes.

#### Exploratory Aim 3

The third exploratory aim is to test the hypothesis that the effects of the FMS intervention will be sustained through week 24.

## Methods

### Study Design

The PLAY study was designed as an attention-matched randomized controlled trial, with the intervention arm receiving FMS instruction and the comparator arm receiving instruction on UPA. The PLAY study sought to recruit 72 child-parent dyads (children 3 to 5 years of age), with the goal of 36 dyads per arm. Parents were targeted to guide the intervention, as parental support, modeling, and co-participation predict children’s engagement in PA [31-34]. Pennington Biomedical Research Center’s Institutional Review Board approved this study (2018-041).

### Sample Size and Power Calculation

The estimated effect size is based on a meta-analysis of FMS interventions (overall effect size *d*=0.39) [35,36]. A group size of 28 dyads/arm was originally planned to provide 80% power to detect an effect size of 0.33 for change in FMS score at week 12 (α=0.05). The research team enrolled and randomized 72 parent-child dyads to allow for a ~22% drop out by week 12 (based on previous studies by the multi-primary investigators), so that there would be at least 28 dyads/group at the end of the intervention (week 12).

### Participants, Eligibility, and Recruitment

Parents of preschool children in a southeastern state of the United States were recruited between May 2019 and August 2019. Parents were recruited using advertisements via local childcare centers, community events, email listserv, and social media. Inclusion criteria for the child were age between 3 and 5 years, physically capable of exercise, and no mobility limitations that would impair PA based on parent report. Child exclusionary criterion was gross motor quotient at “gifted” or “very advanced” based on the Test of Gross Motor Development - 3rd edition (TGMD-3) [37] at baseline screening; the rationale was to avoid ceiling effects in the primary outcome. Inclusion criteria for parents included having a smartphone, willing to download and use the mobile app, having no plans to move during the study period (24 weeks), and having no self-reported mobility limitations that impaired modelling of FMS. Families were compensated US $25 upon completion of the baseline visit, week 12 visit, and week 24 visit, for a total of US $75.

### Randomization and Blinding

Dyads were randomized in a 1:1 ratio to the FMS or UPA condition after all baseline assessments were complete. The statistician (RB) generated a sex-stratified adaptive randomization taking into account baseline FMS. Investigators were blinded. Data assessors were blinded at each assessment visit. Families discovered which condition they were assigned to following the completion of baseline data collections.

### Procedures

After an initial phone screen, eligible preschool children and parents were asked to attend a screening visit. All study visits occurred at a local recreational facility or at the biomedical research center.

#### Screening Visit

Parents received detailed information about the study and provided written informed consent. Eligibility measures were also completed. Parents completed a weekly availability worksheet to identify available times and opportunities to practice lessons with their children using the mobile app at least 5 days per week. The preschool child performed the TGMD-3. TGMD-3 assessments were scored by a trained coder before the baseline visit to determine eligibility. The child was fitted for a hip-worn accelerometer (ActiGraph GT3X+BT), and parents were asked to have their child wear the accelerometer for 24 hours/day for 7 days while the parent completed a wear-time log. The parent was present when the child was fitted for their accelerometer to assist in appropriate placement while at home; parents were also given informational handouts on how to complete the parental log and when and how to take the monitors off.

#### Baseline Visit (Week 0)

The baseline visit was scheduled within 2-3 weeks of the screening visit. Parents returned the accelerometer at the baseline visit. If the child did not have adequate accelerometer wear during the time period, the parent was asked to have the child wear the accelerometer for an additional 7 days. Parents completed questionnaires at the baseline visit, which included parent and child demographics and child self-regulation. A trained research assistant measured the child’s height and weight and conducted the Pictorial Scale of Perceived Movement Skill Competence for Young Children (PMSC) [38,39].

After the collection of baseline data, the trained research assistant revealed randomization from an opaque envelope provided by the statistician. The trained research assistant helped download the PLAY study mobile app onto the parent’s smartphone and selected the FMS version or UPA version based on randomization. The trained research assistant familiarized the parent with the PLAY study mobile app by using the first weekly lesson as an example, showing the videos and explaining the points system.

#### Follow-Up Visits (Weeks 12 and 24)

The parent and child returned for 2 follow-up visits at 12 weeks (end of intervention) and 24 weeks (follow-up) after baseline. The parent was mailed the accelerometer and wear-time log 2 weeks ahead of these visits to provide adequate time for the child to wear the accelerometer for 7 days. Parents returned the accelerometer in-person for each visit. At both follow-up visits, parents completed questionnaires including child self-regulation and acceptability. Anthropometry, PMSC, and TGMD-3 were conducted with the preschool child and in that specific order. A trained research assistant deleted the PLAY study mobile app from the parent’s smartphone at the week 24 visit.

### Intervention

#### App Development

The PLAY app was developed by research scientists with expertise in FMS, developmental psychology, and behavior change and programmed by CyberFision, an app development company. Because the app is web-based, the app can function on a smartphone, tablet, or other mobile internet-enabled device. Parents in both conditions downloaded the PLAY study mobile app. To standardize the appearance and usability of the mobile app across the two conditions, one mobile app was created to house all components of the intervention, with specific features turned on for each condition (ie, parents of the FMS intervention did not see the UPA intervention components and vice versa).

#### Weekly Lessons

Parents in both conditions were instructed to first read the lesson each week, which detailed the purpose and goals for the activity breaks, and then to have their child perform the respective activity break (which was either the FMS or UPA suggested activities). Parents were asked to have their child engage in the activity breaks for at least 12 minutes/day, 5 days a week, for 12 weeks. This resulted in a total of 720 minutes of time directed toward either the FMS or UPA activity breaks. If the child was unable to obtain 12 minutes in one bout, the parent was asked to obtain that total amount within the day (eg, two 6-minute sessions).

#### Reminders and Reinforcement Schedule

One-way SMS or text messages were sent 5 times each week via the mobile app to prompt the parent to read each week’s lesson (1 time/week) and to prompt the parent and child to engage in the activity break (5 times/week). Parents selected times at their screening visit of when they wanted to receive these reminders. Additionally, a reinforcement schedule was built into the PLAY study mobile app by a point system in which the child pressed a star on the screen for each day that they performed the activity break (5 stars available for each week). Parents were encouraged to reward their child with non-food–based rewards such as small toys or non-tangible items such as a high-five. A new lesson was available every 7 days for 12 weeks for 12 lessons total. Lessons were unlocked regardless of the number of activity breaks completed; parents had access to previous lessons but were unable to access future lessons until the respective week. Research assistants also monitored parents’ usage of the app weekly by monitoring whether the parents reported that the child engaged in at least one activity break through the star reporting system. The research assistants telephoned the parent when this information was missing to inquire if there were technical difficulties accessing the app.

#### Fundamental Motor Skill (FMS) Condition

The PLAY app provided parents in the FMS condition with brief instructional lessons, peer modeling videos of FMS, and peer modeling videos of activity breaks to deliver targeted, structured FMS instruction time. The FMS condition was built on a curriculum focused on 6 key FMS (3 locomotor, 3 object control) that were selected to be challenging but developmentally appropriate (hop, throw, slide, kick, jump, catch; each repeated twice). Activity breaks were developed and tested previously in a preschool-age population, demonstrating effectiveness to improve children’s motor skill competence with good adherence [40,41]. Each week’s lesson included 6 videos; 5 videos (approximately 5 seconds in length) showed preschool-aged children performing different aspects of an FMS (eg, stepping with opposition, bending knees). These series of videos were intended to help the parent select components of the FMS to focus on to shape the child’s skill towards mastery [42]. A final video each week (<1 minute in length) showed children demonstrating activities for the activity break, which included activities that reinforced the week’s selected skill. This final video was narrated by children.

For example, on Week 1 Day 1, the parent received a notification to open the app and access the first themed lesson, “Hop.” The parent was asked to read a brief instructional lesson about the targeted FMS (including a description of a proficient “hop”), view the brief videos of the demonstrated FMS and suggested activity break activities, and then engage in a 12-minute activity break designed to help the parent model the skill and provide the preschool child with practice for the targeted FMS. Given the complexity of each FMS, parents were given 5 videos of the subcomponents of completing each FMS and asked to practice the progression that fit the child. For example, for overhand throwing, the child would practice winding up, stepping forward with the opposite foot, following through across their body, and aiming either for distance or accuracy. Therefore, the child and parent had video examples of child peers completing these FMS subcomponents. The goal was to obtain a total exposure of 720 minutes of directed instruction over 12 weeks, a dosage that aligns with prior interventions that effectively improved children’s FMS [35,43].

Behavioral scaffolding [44] and social cognitive theory [44,45] informed the FMS approach. Behavioral scaffolding is a cognitive learning approach to problem solving that allows children to master skills beyond his or her current ability [46,47] and was implemented through video segments of children performing motor skills that increase in complexity. According to social cognitive theory, for modeling to effectively elicit behavior change, the child must undergo a process involving attention, retention, reproduction, and motivation [44,45]. This was accomplished through viewing of peers performing and narrating skills (attention), repeated practice and reminders (retention), replication of modeled activities (reproduction), and perceived competence and video and text files providing intrinsic and extrinsic motivation, respectively (motivation).

#### Unstructured Physical Activity (UPA) Condition

Parents in the UPA (defined as child-led free play) condition had access to the UPA lessons and UPA activity breaks to promote the equivalent amount of UPA time for the child (ie, 720 minutes over 12 weeks). The lessons were adapted to be developmentally appropriate for preschool children using a curriculum previously developed and tested based on social cognitive theory [30]. This comparator arm was selected as it has shown to increase children’s PA levels [30] but does not explicitly target FMS or provide structured lessons to parents on how to model these skills. The following 6 topics were covered: setting goals, making time for child’s free play, being active indoors and outdoors (eg, dancing), reinforcing PA, reducing sedentary behaviors (eg, screen-time), and parental co-participation. The activity breaks provided specific strategies to encourage the child’s UPA (eg, take your child to the park or outside, use your phone alarm to remind your child to be physically active) with an accompanying video narrated by an adult that read aloud the lesson and featured photos depicting images that aligned with the lesson (eg, a photo of a park). There were no children featured in the photos so that the UPA group did not receive any peer modeling. Features of each condition may be found in Table 1.

**Table 1:** PLAY app: features of each condition.

Features	Fundamental motor skills (FMS)	Unstructured physical activity (PA)
Child physical activity	720 minutes of directed instruction on FMS over 12 weeks (12 minutes/day, 5 days/week)	720 minutes of unstructured PA over 12 weeks (12 minutes/day, 5 days/week)
Parent lesson	Parent reads lesson once a week on each targeted FMS (eg, description of a proficient “hop”)	Parent reads lesson on PA support once a week (eg, how to make time for PA)
Peer modeling videos	Parent and child watch video of peers modeling each targeted FMS (eg, a child performing a “hop”)	None
Activity break	Practice, modeling, and reinforcement of each targeted FMS (eg, hopping game)	Activity breaks of unstructured PA (eg, take child to the park, play songs for a dance party)
Push notifications	Once per week to notify of lesson availability; 5 times per week to prompt each 12-minute activity break	Once per week to notify of lesson availability; 5 times per week to prompt each 12-minute activity break
Rewards and reinforcement	Point system reinforcement schedule	Point system reinforcement schedule

### Measures

#### Primary Outcomes

Feasibility was measured as adherence to the mobile app intervention, including objective data on the number of lessons, videos, and activity breaks accessed, and self-reported frequency of interaction with the app through the reinforcement schedule (ie, star reporting system). These data were provided on an ongoing basis by CyberFision. Acceptability was captured at weeks 4, 8, and 12 with an in-app parent survey that assessed satisfaction with the intervention in 4 domains (overall satisfaction, helpfulness, ease of use, and perceived change in child’s motor skills) using a Likert-type scale. The System Usability Scale [48] and a satisfaction scale [49] were completed by the parents at the week 12 and 24 assessment visits. Additional usage data were captured via weekly parent surveys embedded within the app: the context of the activity breaks including the parent’s location, the child’s location, and who participated (weeks 1, 5, 9); barriers to performing the activity breaks and the use of equipment during the breaks (weeks 2, 6, 10); and engagement with the mobile app (weeks 3, 7, 11).

FMS were assessed using the TGMD-3, a direct observation assessment used with children aged 3-10 years [37]. The TGMD-3 is a process-oriented and product-oriented assessment to evaluate FMS performance in 2 subscales: locomotor (run, gallop, one-legged hop, skip, jump, and slide) and ball skills (two-hand strike, one-hand strike, catch, kick, dribble, overhand throw, and underhand throw). By design, these include skills targeted in the intervention. Previous research has demonstrated that the TGMD-3 is a valid and reliable assessment tool [50,51]. Assessments were filmed in an open space and coded by trained research assistants blinded to the purpose of this project. An FMS expert (EKW), who previously attained 99% reliability coding with the author of the TGMD-3 assessment, coded the administrations and was blinded to the experimental condition of the participants.

#### Exploratory Outcomes

To measure PA, the child was asked to wear an Actigraph GT3X+BT accelerometer for 7 days on the right hip, which has been previously validated in preschool children [52]. Minimal wear time was accepted as 4 days at ≥10 hours/day (≥1 weekend day) and 15-second epoch length [53] were used. Cutpoints by Pate et al [52] were used to classify moderate and vigorous PA, and cutpoints by Evenson et al [54] were used to classify sedentary time.

The PMSC [38] was used to examine the child’s perceived movement competence on the 13 skills assessed with the TGMD-3 and took approximately 10 minutes for the child to complete. Previous research has established validity and reliability with this scale in this age range [39].

Self-regulation skills were reported by the parent using the Devereux Early Childhood Assessment, 2^nd^ Edition, which is a 38-item proxy report with good validity and reliability to measure self-regulation and behavioral concerns in children aged 3-5 years [55].

#### Descriptive Characteristics and Potential Covariates

For anthropometry, height and weight were measured using a stadiometer and portable scale, respectively, without shoes, and recorded to the nearest 1.0 cm and 0.1 kg, respectively. A third measurement was taken if the 2 measurements differed by more than 0.5 units. BMI z-score was calculated [56].

To collect sociodemographic data, parents reported information on child and parent age, sex, race/ethnicity, parental education, family structure, childcare or away-from-home care, home environment [57], and household income. To collect health behavior information, parents reported the child’s screen use, sleep, and prior experience of the preschool child and parent with mobile apps. The parent also completed a short questionnaire related to child diet, including intake frequency of specific food groups (high fat foods and sugary drinks), that has been previously validated in this age range [58].

### Statistical Analysis

Intent-to-treat analyses will be used to include all participants with baseline and at least one follow-up value. Additional analyses will be conducted per protocol based on intervention adherence. Final selection of covariates included in the models will be based on model fit statistics, such as AIC.

#### Aim 1

Initial results for feasibility, broken down separately for number of lessons and videos accessed, activity breaks accessed, and frequency of interaction, will be expressed using contingency tables. Rates, based on a generalized linear model with a Poisson distribution, will be used to both adjust for covariates and compare the 2 versions of the app (FMS vs UPA). Each of the 4 domains of acceptability will be treated independently and considered as a continuous response, analyzed with a linear model for covariate and app effects.

#### Aim 2

FMS results will be estimated using a linear mixed effect model with the baseline and follow-up scores at week 12 (end of intervention) and week 24 (end of study) as the outcome. This model will use random effects to account for the correlation in a participant over time. Results from the model will be reported as least square means, with *P* values based on *t* tests.

#### Exploratory Aims

PA levels, perceived movement competence, and self-regulation will be analyzed similarly as for Aim 2. FMS will be tested as a potential mediator and moderator. Mediations will investigate how the covariates affect the relationship between the condition and each outcome variable (for the exploratory analyses, these include PA levels, perceived movement competence, and academic readiness or self-regulation). Mediation analyses will be applied using both structural mean models and principal stratification [59].

Sustained effects will be estimated using a similar linear mixed model as described in an earlier section. However, time will be considered a continuous covariate in order to estimate the rate of sustained effect. This allows the interaction of app and time to test for rate differences between the 2 apps on outcomes of interest through the follow-up at weeks 12 and 24 (separate models conducted for each dependent variable measured continuously over time: FMS, PA levels, perceived movement competence, and self-regulation).

## Results

This project was funded from September 2018 through 2021 by the Eunice Kennedy Shriver National Institute of Child Health and Human Development of the National Institutes of Health. Pennington Biomedical Research Center’s Institutional Review Board approved this study in August 2018. Recruitment occurred between May 2019 and August 2019. Accordingly, data collection happened from May 2019 through February 2020, including all baseline, week 12, and week 24 visits. To date, the project team has completed data collection on 69 parent-child dyads and is currently analyzing the primary and exploratory outcomes. Results of the current study are expected to be published in 2021.

## Discussion

PLAY is a parent-targeted, theoretically grounded, mobile-based intervention designed to teach parents how to model, support, and guide their preschool-aged children’s FMS competence. The scientific premise for the protocol is based on: (1) evidence showing that children must establish a minimal level of FMS proficiency to continue participating in PA opportunities as they age [4-8]; (2) longitudinal data linking FMS proficiency in childhood with higher levels of PA and lower adiposity 4 y later [15] and into adolescence [12], including an FMS intervention that increased preschoolers’ PA [60]; (3) the lack of published mHealth interventions that specifically focus on parents of preschool-aged children or on PA promotion or FMS development; (4) the mixed results of prior mHealth interventions, which are at least partially attributable to previous efforts failing to fully capitalize on behavior change theory and maintain treatment intensity; and (5) the desire of parents to have family-focused interventions that are convenient to access [61].

Unlike other interventions that are primarily in childcare settings [11,62], this intervention utilizes parents to deliver the FMS curriculum, as parents serve an important role to enable early PA behaviors [63]. Previous work in low-income populations found that some of parents’ PA was related to their preschool children’s PA, providing more evidence that these behaviors could be modeled from parent to child [64]. Given the ubiquity of digital devices in parents’ and children’s daily lives [21], this study will leverage this existing screen-time and mobile app use to promote FMS development and healthy development in children.

The use of a mobile app to deliver the intervention offers the unique opportunity to distribute evidence-based content in a succinct and easily accessible manner to parents. This mode of delivery directly to the hands of the parent offers many advantages compared to receiving this instruction at in-person classes or health clinics, reducing barriers such as transportation, time commitment, and other family priorities [65]. The app offers ecological validity, so the child is practicing FMS and engaging in PA in the real-world setting of their own home rather than in a research laboratory. Further, the curriculum delivered on the mobile app is grounded in behavior change techniques, such as behavioral scaffolding and peer modelling, whereas many other mobile apps incorporate few behavior change techniques to shape behaviors [66]. Apps also offer the opportunity to incorporate real-time feedback through wearable devices, such as step counters and accelerometers, which are growing in popularity and acceptability by parents [67] but may or may not be acceptable or feasible for preschool-aged children.

One of the key strengths of using a mobile app is the opportunity for dissemination and implementation (ie, scaling up the intervention). The mobile app may be readily updated from parents’ feedback and thereby adapted in a timely manner. This adaptability allows the study to bypass challenges in dissemination and implementation commonly presented in school and health care interventions, such as structural and personnel limitations. For example, the PLAY study mobile app delivers lessons that require little to no equipment and provides lessons directly to the parent and child. These two components decrease the potential for organizational challenges, along with hiring and training personnel to implement the intervention. Additionally, mobile apps allow accessibility to families and individuals living in rural areas where access to appropriate curriculum and health-enhancing activities is limited. A FMS curriculum delivered on a smartphone app is an innovative and potentially acceptable way to create a social learning environment that can benefit young children and their parents from their homes.

There are potential limitations to the PLAY study. The parents were provided with videos that demonstrate each level of FMS progression divided into skill components and expected to determine their child’s ability and adjust the activities for their child’s current FMS level. An important consideration is that previous studies indicate parents do not provide a valid report of their child’s FMS [68,69], which may lead to parents delivering content or curriculum that is not developmentally appropriate for their child. While the study outcome data on FMS were collected using a valid and reliable test and scored by a trained expert, future iterations may share these data directly with the parent to assist their intervention delivery.

One challenge is potential malfunction of the mobile app that can influence delivery and lesson implementation during the trial. The research study team conducted usability testing prior to use and also contacted parents with technical support if they did not engage with the mobile app (via reporting participation) at least once per week. This communication was intended to allow any mobile app errors to be found in real-time and quickly addressed. Another limitation is a concern in app research, which is differentiating between adherence to protocol (ie, completing the activity breaks) and engagement with the mobile app (ie, opening and utilizing the mobile app) [70]. This study used mobile app data (eg, viewing videos) as objective measurements of utilization of the app, but adherence to the protocol was limited to parent-report based on the family selecting stars each week to indicate completed activity breaks. Finally, as these data were collected in the southeastern United States, generalizability of the results of this feasibility study will be limited to this region. Future work will be needed to assess the feasibility in different regions, as well as to examine the effectiveness of this intervention in different subgroups (eg, sex, race/ethnicity, income level).

Overall, parents and preschool children spend much of their time using screens, and this existing screen-time may provide an opportunity to deliver beneficial and developmentally appropriate content to enhance FMS proficiency in young children. The PLAY study examines the feasibility and preliminary effectiveness of a mobile app and parent-led curricula to promote FMS proficiency for preschool children. If found to be effective, the app has the potential for wide-scale dissemination to parents of preschoolers and to provide a model for the utilization of mobile apps to promote young children’s motor skill development.

## Abbreviations

FMS: fundamental motor skills
mHealth: mobile health
PA: physical activity
PLAY: Promoting Lifelong Activity in Youth
PSMC: Pictorial Scale of Perceived Movement Skill Competence for Young Children
TGMD-3: Test of Gross Motor Development – 3rd edition
UPA: unstructured physical activity
